# Diagnosing major depressive disorder and substance use disorder using the electronic health record: A preliminary validation study

**DOI:** 10.1016/j.xjmad.2023.100007

**Published:** 2023-06-22

**Authors:** Vinod Rao, Sylvia Lanni, Amy M. Yule, Maura DiSalvo, Mira Stone, Amy F. Berger, Timothy E. Wilens

**Affiliations:** a Department of Psychiatry, Massachusetts General Hospital, 55 Fruit Street, Boston, MA 02114, USA; b Department of Psychiatry, Boston Medical Center, 801 Massachusetts Avenue, Boston, MA 02118, USA

**Keywords:** Depression, Substance use, Electronic health record, Methods, Validation

## Abstract

**Background:**

One mechanism to examine if major depressive disorder (MDD) is related to the development of substance use disorder (SUD) is by leveraging naturalistic data available in the electronic health record (EHR). Rules for data extraction and variable construction linked to psychometrics validating their use are needed to extract data accurately.

**Objective:**

We propose and validate a methodologic framework for using EHR variables to identify patients with MDD and non-nicotine SUD.

**Methods:**

Proxy diagnoses and index dates of MDD and/or SUD were established using billing codes, problem lists, patient-reported outcome measures, and prescriptions. Manual chart reviews were conducted for the 1-year period surrounding each index date to determine (1) if proxy diagnoses were supported by chart notes and (2) if the index dates accurately captured disorder onset.

**Results:**

The results demonstrated 100% positive predictive value for proxy diagnoses of MDD. The proxy diagnoses for SUD exhibited strong agreement (Cohen's kappa of 0.84) compared to manual chart review and 92% sensitivity, specificity, positive predictive value, and negative predictive value. Sixteen percent of patients showed inaccurate SUD index dates generated by EHR extraction with discrepancies of over 6 months compared to SUD onset identified through chart review.

**Conclusions:**

Our methodology was very effective in identifying patients with MDD with or without SUD and moderately effective in identifying SUD onset date. These findings support the use of EHR data to make proxy diagnoses of MDD with or without SUD.

## 1 Introduction

Depressive disorders, particularly major depressive disorder (MDD), impact millions of individuals in the United States. Research suggests that over 20% of adults in the United States will experience MDD at some point in their lifetime [1]. Young adulthood represents a particularly vulnerable developmental period for the onset of MDD, as prior work has shown the median onset age for unipolar depression to be in the mid to late 20 s, with 37% of onset of depressive disorders occurring by age 25 [2], [3]. The sequelae of MDD are wide-ranging and include impairment in school and work [4], poor health [5], [6], mortality [7], and other psychiatric disorders [8], [9], [10].

Individuals with MDD exhibit particular risk for the development of substance use disorder (SUD). A recent review and meta-analysis by Hunt et al. (2020) found that 25% of adults with MDD have a co-occurring SUD [11]. Furthermore, in 2020, over 600,000 adolescents (2.7%) in the United States reported a co-occurring depressive disorder and SUD in the past year, and adolescents with a depressive disorder were significantly more likely than those without to have used illicit drugs (28.6 vs. 10.7%) [4]. The combined impairment of daily functioning caused by MDD and SUD, as well as the associated medical costs for treatment pose a substantial societal burden, particularly for young people [12], [13], [14], [15]. Indeed, MDD when comorbid with SUD is associated with greater vulnerability to additional psychopathology, increased rates of and more severe depressive episodes, and increased rates of suicide attempts in comparison to MDD alone [16].

### 1.1 MDD treatment and subsequent SUD

In studies examining the impact of treating juvenile-onset MDD on the later development of SUD, preliminary findings suggest that treatment predicts lower rates of the subsequent development of SUD across different treatment modalities [17], [18], [19], [20]. For example, Curry et al. (2012) found that the combined treatment of 12- to 18-year-old patients with Cognitive Behavioral Therapy and fluoxetine significantly reduced the rate of subsequent drug use disorders after 5 years [19]. Similarly, Carrà et al. (2019) found that youth and adults treated for MDD were significantly less likely to report non-medical use of prescription opioids than individuals who were not treated for MDD [18]. Nonetheless, existing studies examining depression treatment and SUD vary greatly in methodology, with studies differing in lengths of follow-up, study design, and sample size [21], [22], [23].

Given the variation in methodology between studies, the relationship between the treatment of depression and its impact on the subsequent development of SUD outcomes remains unclear and merits further examination. More rigorous approaches such as randomized controlled trials (RCTs) are not viable in studying the natural course of MDD and subsequent SUD, as the ethical dilemma of withholding treatment necessitates the use of systematically ascertained, longitudinal, naturalistic data to examine the trajectory of patients’ behavioral health outcomes such as SUD in context to treatment of MDD.

### 1.2 The electronic health record

One compelling mechanism to examine the impact of disorders and their treatments on subsequent sequelae and/or disorders is to utilize the longitudinal electronic health record (EHR). EHRs have the potential to examine large groups of heterogeneous patients receiving various treatments naturalistically over time. Multiple outcomes, including changes in behavioral health symptoms and new diagnoses, as well as intervening factors, such as sociodemographics, past diagnoses, and various treatment exposure, influencing these outcomes can be examined. The use of the EHR in the routine care of patients has grown exponentially since the 1990 s [24] with a commensurate, albeit lagged, increase in research utilizing EHRs [25].

As the EHR is designed to facilitate clinical care and administrative functions for individual patients, analyzing data across patients requires significant transformation into usable formats [26]; however, inconsistent documentation practices across clinicians hinder this process [27]. Moreover, omission of diagnoses, particularly in behavioral health settings, limits the utilization of the data [28], [29]. The recent emergence of measurement-based care and patient-reported outcome measures (PROMs) appears to have enhanced the diagnostic sensitivity of EHRs through both clinical interviews by trained clinicians and diagnoses derived from validated screening tools [30], [31], [32], [33], thus enhancing the complete EHR's advantage over claims-based databases, which lack these patient-reported data.

Studies examining the diagnostic accuracy of EHR data present mixed results. A review by Davis and colleagues (2016) assessed how official clinical documentation in the patients’ clinical chart of various diagnoses compared to diagnoses recorded in research or administrative databases [34]. When examining the accuracy of routinely collected mental health diagnoses, researchers identified large amounts of variation in the positive predictive value (PPV) and agreement within different diagnoses. For example, while substance misuse and anxiety disorders exhibited a relatively low median PPV in the reviewed studies (<60%), even the disorders exhibiting higher PPV such as schizophrenia only had a median of 75% PPV. Similarly, a review by Larvin and colleagues (2019) assessed case-finding for common mental disorders in routinely collected data [35]. The researchers found that diagnostic codes for depressive disorders exhibited low sensitivity, not exceeding 0.38. The included studies varied in type of reference standard, including diagnostic interviews [36], [37], [38], self-reported questionnaires [39], [40], [41], researcher and clinician reviewed EHR [40], [42], physician questionnaire [43], and case-control design [42].

Though most studies of depression diagnoses using EHR data utilize diagnostic codes [37], [38], [39], [41], [42], [43], [44], a minority of research has examined the accuracy of combined diagnostic and prescription codes [40], [44], the accuracy of prescription codes alone [36], [37], [43], [44], and the accuracy of free text (i.e., the primary diagnosis recorded by primary care providers in a patient’s chart) [45]. More accurate case definitions for common mental health disorders comprised diagnostic and prescription codes together (sensitivity>0.9) [40]. For existing studies of prescription codes of depressive disorders, sensitivity is highly variable, ranging from 0.14 to 0.69. Finally, the sensitivity of free text for depressive disorders is also variable, ranging from 0.20 to 0.58 [35], [45]. Interestingly, though PROMS have been utilized as a reference standard for analyses of EHR accuracy [46], to our knowledge, no studies have examined the diagnostic accuracy of PROMs or other forms of measurement-based care reported on the EHR.

Given the variation in study methodologies using EHR data and the reported low sensitivity of these analytic strategies, a comprehensive approach to account for the inconsistencies in EHR data is greatly needed using available elements in the EHR. Clear data extraction rules and variable construction linked to psychometrics validating their use are needed to prevent the inadvertent omission or commission of pertinent data. We therefore propose a methodologic framework for examining EHR data with our initial example to evaluate the impact of treating MDD on the development of a non-nicotine SUD. We will evaluate this approach’s potential to identify individuals with MDD and co-occurring MDD and SUD and test the psychometrics of this approach through interrogation of the EHR.

## 2 Methodological proposal

### 2.1 Study setting

We propose a methodological framework for examining EHR data from patients evaluated and treated in medical, surgical, and behavioral health services at a diverse urban and rural hospital system in the northeastern United States. Our initial focus was on MDD and SUD. This data was extracted as part of a broader research study examining how treating psychopathology impacts the development or worsening of SUD. As such, the current framework focuses on patients aged 16–30 years, detailing how to identify patients with MDD who subsequently developed non-nicotine SUD. Due to the methodology of the broader research study and in order to affirm that all included patients had at least one encounter with behavioral health, the original sample was ascertained from four behavioral health outpatient clinics in the hospital system. However, EHR encounters were examined from any medical, surgical, or behavioral health encounter in the wider EHR system.

### 2.2 Exclusion criteria

As this was conducted as part of a research study examining MDD and SUD, we excluded patients with diagnoses of bipolar disorder (ICD-10 codes: F30.*-F31.*) or primary psychotic disorders (ICD-10 codes: F20.*-F29.*) as these disorders interfere with the phenotype of depression for the sake of this study. For example, prior EHR analyses have found that the inclusion of diagnostic codes for bipolar disorder reduces the PPV for MDD diagnosis [46]. In addition, we did not include patients who met criteria for SUD prior to or during an initial major depressive episode (MDE), as our research question focuses on the *subsequent* development of SUD after MDD onset.

### 2.3 MDD diagnosis

#### 2.3.1 Billing codes

The presence of MDD was determined by extracting information from multiple areas in the EHR to best account for the likelihood of omission of coding or variation in diagnostic and billing codes. We first identified individuals with likely depressive disorders based on diagnostic billing codes (ICD-10 codes: F32.*-F39.*). For individuals with these codes, diagnoses were made clinically in the course of routine practice. Problem lists for each patient were also extracted to improve identification of MDD, as research suggests that relying only on encounter data to identify psychiatric conditions may under-capture these conditions [47].

#### 2.3.2 PROMS

In addition, positive response for depression criteria on the Patient Health Questionnaire 9 (PHQ-9), a component of PROMs administered as standard clinical care, was also utilized as a proxy for MDD diagnosis [48]. In the present hospital system, the PHQ-9 is routinely offered to all patients at intake and periodically thereafter in behavioral health clinics and adult primary care. Prior research suggests that recording depressive symptoms rather than MDD as a psychiatric diagnostic category may be an increasing trend among physicians as it is less stigmatizing and better captures milder symptomology [49], [50]. Moreover, using PROMs scores as a proxy for MDD diagnosis may account for differences in provider billing choices. Data have also established that depressive disorders are often underdiagnosed in primary care, and that PROMs may be used as alternative markers of MDD [39], [51]. We used a cut-off threshold score of 13 or greater on the PHQ-9 given its high sensitivity and specificity exhibited in prior research [52].

#### 2.3.3 Therapeutics

Medications typically used for treating MDD as defined by the literature [53] were also utilized as a proxy for MDD diagnosis to identify an index date for the onset of MDD if a depressive disorder diagnosis was later documented in the patient’s chart. Though medication does not indicate with certainty the presence of a particular condition, that a depressive disorder is later documented increases confidence that the medication was prescribed to improve symptoms of MDD that a patient was experiencing at that time. Registration of antidepressant prescriptions has been purported to be a better indicator of clinician recognition of patient MDD than diagnostic codes [36], in part related to cases of comorbidity in which practitioners may not code all relevant diagnoses to save time [54].

A team of experienced board-certified psychiatrists also reviewed the medications that were used as a proxy for the diagnosis of MDD (See Table 1 for included antidepressants and minimum effective dose information). Medications alone were not sufficient to make a diagnosis of MDD when medications were clearly used to treat symptoms of non-MDD conditions such as anxiety, eating disorders, or OCD. As such, patients who were prescribed anti-depressant medication and received a relevant diagnostic code for a non-MDD condition prior to a depressive disorder diagnosis (ICD-10 codes: F41.0-F41.1, F41.9, F42.2, F42.8-F43.0, F43.10, F43.12, F43.21-F43.23, F43.9, F50.00-F50.02, F50.2, F50.9, F53.0, O99.345, O99.891) did not receive a re-indexed MDD date as a result of the prescription.

**Table 1:** Included Depression Medications and Minimum Effective Dose.

Depression medication proxies^a^	
Medication	Minimum effective dose (mg/d)
Citalopram	20
Escitalopram	10
Fluoxetine	50
Paroxetine	20
Sertraline	50
Vilazodone	20
Vortioxetine	10
Amitriptyline	75
Clomipramine	75
Doxepin	75
Imipramine	75
Nortriptyline	75
Trimipramine	75
Desipramine	200
Maprotiline	200
Protriptyline	41
Isocarboxazid	30
Phenelzine	45
Tranylcypromine	20
Selegiline	41
Selegiline (TD)	9
Duloxetine	60
Venlafaxine	75
Desvenlafaxine	50
Levomilnacipran	40
Mirtazapine	30
Amoxapine	400
Nefazodone	300
Reboxetine	8
Bupropion	150
Esketamine	Any Rx

a Adapted from: [53]

#### 2.3.4 Integrative diagnosis

All patients classified as having MDD must have met at least one of the above-listed criteria of diagnostic code, PROMs or PHQ-9 endorsement, and/or medication used for MDD with subsequent evidence of MDD. For patients who reached multiple criteria, the MDD index date was the date at which the earliest criterion was reached.

### 2.4 SUD diagnosis

In the current analyses, nicotine and tobacco use and use disorders were understood as separate from other SUDs given their treatment as such in prior literature [55], [56], [57]. Because prior analyses have indicated that nicotine use may increase chances for the subsequent development of SUD [55], [56], nicotine and tobacco use are to be included in later analyses as a potential mediator/moderator of SUD development following MDD diagnosis but were not examined in the current sample.

#### 2.4.1 Billing codes

As with MDD proxy diagnoses, identification of non-nicotine SUDs was determined by extracting information from multiple areas in the EHR to best account for the likelihood of errors or variation in diagnostic and billing codes. We first identified individuals with SUD based on diagnostic billing codes (ICD-10 codes: F10.*-F16.*, F18.*-F19.*). For individuals with these codes, SUD diagnoses were made clinically in the course of routine practice. As with MDD identification, problem lists for each patient were also extracted to improve identification of SUD.

#### 2.4.2 PROMs

In addition, positive responses for SUD criteria on PROMs, including the Alcohol Use Disorders Identification Test (AUDIT-C) [58] and the Tobacco, Alcohol, Prescription Medication, and Other Substance Use tool (TAPS) [59] served as a proxy for SUD diagnosis. Although elevated scores on SUD screening tools are not equivalent to formal SUD diagnosis, our inclusion of PROMs as proxy measures of SUD was intended to account for the underdiagnosis of SUDs in clinical practice evident in prior research [60], [61]. As such, validated cut-off scores for included screeners were used to capture suspected SUD.

##### 2.4.2.1 AUDIT-C

The AUDIT-C is a 3-item alcohol screening questionnaire, with scores ranging from 0 to 12 and higher scores indicating greater likelihood of alcohol use disorder (AUD). Our diagnostic threshold for this questionnaire was a total AUDIT-C cutoff score of 8. This cutoff has been found to have a specificity of 99% in identifying heavy drinking and/or active alcohol abuse or dependence [58].

##### 2.4.2.2 TAPS

The TAPS screens for tobacco, alcohol, illicit drugs, and non-medical prescription drug use, starting with 4 items and then reflexing to up to 13 items depending on patient endorsement of specific drug use. Scores on the reflexed questions, also known as the TAPS-2, range from 0 to 2 + , with zero indicating no use of a particular substance in the past 3 months and 2 indicating high-risk level for a particular substance. The recommended cutoff for identifying SUD is 2 + , with the tool having an adequate sensitivity (>70%) for risky tobacco, alcohol, and marijuana use at this cutoff [59].

#### 2.4.3 Therapeutics

FDA-approved medications for the treatment of SUD were also utilized as a proxy for SUD diagnosis to identify an index date for the onset of SUD if a relevant SUD diagnosis was later documented in the patient’s chart. The medications affecting SUD diagnoses were reviewed by a team of experienced board-certified psychiatrists, and included the following: naltrexone, acamprosate, disulfiram, methadone, and buprenorphine. Medications with indications in prescription notes for cancer, palliative care, or severe/excessive/chronic pain were excluded.

#### 2.4.4 Integrative diagnosis

All patients classified as having SUD must have met at least one of the criteria listed above. For patients who reached multiple criteria, the SUD index date was the date at which the earliest criterion was reached.

### 2.5 Validation methods

To examine the validity of our MDD and SUD diagnoses, we randomly selected 50 patients who met proxy criteria for MDD and/or SUD and interrogated EHR encounter notes for each patient to determine the accuracy of the diagnoses. All the patients in our validation sample met proxy criteria for MDD. To effectively examine the accuracy of our SUD proxy diagnoses, when identifying patients for the validation analyses, we stratified patients by eventual SUD status (e.g., individuals who subsequently met SUD proxy criteria versus those who do not). We then stratified patients by sex at birth to get an equal representation of males and females, as symptomology for psychiatric disorders can vary between sexes and may impact clinician documentation of diagnoses [62], [63]. Twelve to 13 patients were then randomly selected from each stratification group. Thus, our final validation sample included 25 patients with MDD who developed SUD and 25 patients who did not develop SUD, with half of each of these groups being male at birth.

For both MDD and SUD, the reference standard to which proxy diagnoses were compared was systematic review of chart notes, given the use of similar reference standards in prior work [40], [42]. Thus, our validation procedure aimed to compare the accuracy of the presence and onset of the identified proxy diagnoses against provider chart notes as opposed to recorded diagnoses in the patient chart.

Two trained research assistants reviewed chart notes 6 months before and 6 months after the date on which each participant met criteria for a proxy definition for MDD. A validation range of 1 year was chosen given the variation in frequency at which patients are seen in the hospital clinics and the use of similar index periods in the literature [64], [65].

Because the focus of the current study is on which participants may go on to develop a SUD rather than patients with pre-existing SUD, study staff also examined the chart notes within the 3 months before and after the MDD index date to rule out the presence of a pre-existing SUD or substance-induced mood disorder. Because symptoms for substance-induced mood disorders never precede the onset of substance use and typically subside within a month of cessation of intoxication or acute withdrawal [66], 6 months was used as a time period to determine whether a SUD was already present at the time of the MDD index date.

For individuals who eventually received a SUD diagnosis according to our proxy definitions, after looking at the MDD index date and confirming that no SUD concern was present at that time, study staff examined chart notes 6 months prior to and post the date on which each participant met criteria for SUD. For individuals who did not eventually receive a SUD diagnosis according to proxy definitions, after looking at the MDD index date and confirming that no SUD concern was present at that time, study staff examined chart notes 6 months prior to the date of the final data extraction to confirm that no SUD concern existed within this timeframe. Because all study staff were aware of the final data extraction date, blinding of staff to patient stratification group was not possible.

The initial decision on the presence of a proxy diagnosis was reviewed by a team of experienced board-certified general and addiction psychiatrists, and discrepancies were resolved.

#### 2.5.1 Data analysis

We calculated sensitivity, specificity, positive predictive value (PPV), and negative predictive value (NPV) using the numbers of true/false positive and true/false negative proxy results [67]. Ninety-five percent confidence intervals were calculated using the Wilson score interval method [68]. Cohen’s kappa (κ) with 95% confidence intervals calculated through bootstrapping was also computed to examine agreement between proxy SUD diagnoses and SUD diagnoses identified through manual chart review [69].

## 3 Results

The validation sample (N = 50) had a mean age of 23.5 years (SD=4.32). Regarding race and ethnicity, 78% (N = 39) of patients identified as White, 12% identified as Black or African American (N = 6), 2% identified as Asian (N = 1), and 8% declined to provide their race (N = 4). Four participants (8%) identified as Hispanic or Latinx. Due to our stratification, 50% (N = 25) of the sample was male at birth. Of the entire sample, the source of the MDD index date was billing codes for 72% of patients (N = 36), medication prescriptions for 22% of patients (N = 11), and PROMs for 6% of patients (N = 3). Of the 25 patients who screened positive for SUD, the source of the SUD index date for 24 patients (96%) was billing codes, while the source for the remaining patient was PROMs (AUDIT). No patients in the validation sample screened positive for SUD based on TAPS scores.

Because all patients in the sample screened positively for MDD, only PPV could be calculated for this diagnosis. Our proxy diagnoses for MDD exhibited a PPV of 100%.

The sample was designed so that half of the included patients (N = 25) screened positive for SUD while the other half of patients screened negative. Our proxy diagnoses for SUD had 92% sensitivity, 92% specificity, 92% PPV, and 92% NPV (all 95% CIs: 0.774, 0.993) (Table 2). Cohen’s κ for SUD diagnoses identified through proxy criteria versus through manual chart review indicated strong agreement between the information sources (κ = 0.84, 95% CI: 0.72, 0.94) [69]. Of note, these values only account for the presence of SUD and not for the accuracy of the index date for SUD. Our validation identified two false positive and two false negative cases. In the two false positive cases, patient charts contained diagnostic billing codes for substance use that did not indicate any functional impairment (e.g., medical marijuana use, cannabis use). In the two false negative cases, patient substance abuse was documented in their respective chart notes, but this information was not reflected in any other area of the EHR and thus was missed by our data extraction. Of the accurately identified patients (N = 23), the most represented types of SUD at the index date included alcohol (N = 10) and cannabis-related disorders (N = 9), with only 2 patients’ SUD index dates being based on stimulant use disorder. An additional two patients received a positive SUD screen for AUD and cannabis use disorder and for polysubstance dependence, respectively.

**Table 2:** SUD validation results.

	Has the condition	Does not have the condition
Screened positive	**a**. True positive= 23	**b**. False positive= 2
Screened negative	**c**. False negative= 2	**d**. True negative= 23

The proxy rules were less reliable at documenting the onset of SUD, as 16% (N = 4) of the 25 patients who screened positive for SUD had inaccurate SUD index dates that were recorded after the 6-month window following SUD onset. For 2 of the 4 patients with inaccurate SUD index dates, SUD was documented in the chart prior to the index date in visit notes from external providers. Because these notes were not generated within the hospital system, they were not included in the original data extraction. Of these two patients, one patient’s SUD was documented as starting 1 year prior to our SUD index date, and the second patient’s SUD was documented as starting at least 10 years prior to our index date. For the other two patients, chart notes from their respective SUD index dates contain SUD diagnoses labeled as in remission. Though no additional information was provided for either of these patients to allow for an estimate of when substance use began or became problematic, the inclusion of the “in remission” qualifier indicates substantial prior substance use that was not captured through our proxy definitions.

## 4 Discussion

The current paper details a preliminary validation of our methods for identifying diagnoses by curating a variety of information through systematic extraction of the EHR and by establishing diagnoses of MDD and co-occurring MDD and SUD by proxy. Our methodology, integrating diagnostic billing codes, EHR problem lists, medication prescriptions, and PROMs, was highly effective in identifying patients with MDD and co-occurring MDD and SUD. Our methodology was moderately effective in identifying the accurate onset date of SUD in patients with MDD.

The results of this validation study underscore the importance of integrating a variety of information into proxy diagnoses when attempting to capture diagnostic information from the EHR. In prior studies of systematic EHR chart reviews, substance use in particular exhibits low PPV when relying solely on diagnostic billing codes to identify positive screens [34]. Through the integration of multiple information sources within the EHR, we were able to achieve high PPV and NPV in comparison to other existing studies [70], [71].

To our knowledge, this is the first study to utilize PROMs as a proxy diagnosis for MDD and SUD. Though few of the randomly selected patients in this validation sample were identified as having either MDD (N = 4) or SUD (N = 1) based on PROMs alone, for each of these patients PROMs accurately identified the presence of the respective psychiatric condition when validated against chart notes. Future studies examining the diagnostic accuracy of PROMs alone as well as the diagnostic accuracy of PROMs in combination with other information sources are needed to further elucidate the utility of PROMs in similar EHR data extractions.

The high sensitivity, specificity, NPV, PPV, and Cohen’s κ of our data highlight the validity of using systematically extracted and readily available variables to establish the presence or absence of psychiatric and substance use diagnoses for clinical and research purposes. Moreover, this method also preliminarily validates modestly the onset of the index case of MDD or SUD. While imperfect, the determination of diagnoses and their onsets has tremendous utility in examining large clinical electronic databases for both cross-sectional and longitudinal data with high clinical and public health relevance. For example, in the current data, we have the capacity to examine mediators and moderators available in the EHR related to the subsequent development of SUD in patients with MDD.

The results of this study must be interpreted in the context of substantial methodological limitations. Our study was conducted in a relatively small sample of individuals in one hospital system necessitating replication in larger samples representing more diverse geographic and demographic characteristics. The time-intensive nature of our chosen reference standard, manual review of patient chart notes, required use of a smaller sample size, limiting the potential generalization of our results. Nonetheless, potential limits of generalizability introduced through the modest sample size were partially addressed by the randomized selection of patients. Furthermore, the calculated Cohen’s κ indicates strong agreement between our proxy diagnoses and provider chart notes while still taking into consideration the limited sample size [69]. Regarding other sample limitations, our sample was also relatively racially and ethnically homogenous, with 78% of patients identifying as White. Although this racial distribution is reflective of the population served by the hospital system, future research dedicated to groups underrepresented in the current study is of pressing need. Finally, our sample consisted only of patients aged 16–30 years. This inclusion criterion was implemented to capture the period of greatest risk for depression onset, as determined by prior research [2], [3]; however, replication of the current methodology in a wider range of patients would improve the study’s generalizability.

An additional limitation of the current study is the small number of patients inaccurately identified as having or as not having SUD. In two instances, providers used diagnostic billing codes for substance use that did not indicate any functional impairment. As such, errors in proxy diagnoses resulted from provider differences in the documentation of substance use and SUD. These errors may be improved by incorporating natural language processing of chart notes into proxy diagnoses [72]. A particular strength of the current study is its accurate identification of the presence or absence of SUD in various patients in comparison to prior literature [34]. Nonetheless, the proxy diagnoses less reliably identified the onset of SUD. Several possibilities may account for this error. Patients may receive care for SUD from providers outside the EHR system, which would prevent these encounters from being captured [73]. Further, providers may be hesitant to diagnose a patient with SUD or may not have sufficient time in a visit to assess substance use. In addition, although this validation approach would not have captured this, patients may be hesitant to disclose substance use due to fear of stigma [74], [75] and may wait until becoming more familiar with a provider. Another limitation is the inability to assess subthreshold symptomology with the given proxy diagnoses. Research has established a significant likelihood for subthreshold psychiatric conditions to develop into full symptomology [76]. However, the EHR currently contains no systematic documentation of subthreshold conditions. When attempting to determine the progression of MDD and the subsequent development of SUD, capturing problematic substance use prior to the development of a full SUD may better elucidate the pathways linking these disorders.

### 4.1 Conclusions

Despite these limitations, the current study indicates that integrating billing codes, problem lists, medication prescriptions, and PROMs into EHR data extractions appears to be a feasible and valid method of determining patient MDD and SUD diagnoses, and to a lesser extent, the index onset of these disorders. Using similar techniques for research moving forward can enhance studies using naturalistic data while mitigating the time and burden associated with manual chart reviews. These techniques may also be utilized to create EHR-embedded decision support tools to facilitate evidence-based treatment and thereby improve the quality of care. Further replication in larger samples in more diverse medical systems is necessary.

## Funding

Dr. Wilens and Dr. Yule have reported funding for this work from 10.13039/100000026NIDA grant UH3DA050252.

## Declaration of Competing Interest

The authors declare the following financial interests/personal relationships which may be considered as potential competing interests; Timothy Wilens reports financial support was provided by National Institute on Drug Abuse. Amy Yule reports financial support was provided by National Institute on Drug Abuse, the Doris Duke Charitable Foundation’s COVID-19 Fund to Retain Clinical Scientists collaborative grant program through support from the John Templeton Foundation, and the National Center for Advancing Translational Sciences, National Institute of Health, through the Boston University Clinical and Translational Science Institute. She also has funding for clinical program development from the Jack Satter Foundation. Timothy Wilens reports relationships with US Minor/Major League Baseball, Gavin Foundation and Bay Cove Human Services that include: consulting or advisory. Amy Yule reported relationships with Gavin House and Bay Cove Human Services and the American Psychiatric Association’s Providers Clinical Support System Sub-Award that include: consulting or advisory. Timothy Wilens has a licensing agreement with Ironshore (BSFQ Questionnaire) and 3D Therapeutics. Corresponding author co/edited books: ADHD in Adults and Children (Cambridge University Press), Straight Talk About Psychiatric Medications for Kids (Guilford Press), An Update on Pharmacotherapy of ADHD (Elsevier Press) -T.W.
